# Stability and Antibiofilm Efficiency of Slightly Acidic Electrolyzed Water Against Mixed-Species of *Listeria monocytogenes* and *Staphylococcus aureus*

**DOI:** 10.3389/fmicb.2022.865918

**Published:** 2022-05-12

**Authors:** Pianpian Yan, Ramachandran Chelliah, Kyoung-hee Jo, Vijayalakshmi Selvakumar, Xiuqin Chen, Hyeon-yeong Jo, Deog Hwan Oh

**Affiliations:** ^1^Department of Food Science and Biotechnology, College of Agriculture and Life Sciences, Kangwon National University, Chuncheon, South Korea; ^2^SeouLin Bioscience Company and Limited, Seongnam-si, South Korea; ^3^Kangwon Institute of Inclusive Technology (KIIT), Kangwon National University, Chuncheon, South Korea; ^4^Saveetha School of Engineering, (SIMATS) University, Sriperumbudur, India

**Keywords:** SAEW, mixed-species, antimicrobial activity, biofilm, shelf life

## Abstract

In the natural environment, most microorganisms live in mixed-species biofilms, in which the metabolism and growth of organisms are different from that in single-species biofilms. Adhesive bacteria and their biofilms on the surface of food processing equipment are the sources of cross-contamination, leading to the risk for humans. Slightly acidic electrolyzed water (SAEW) has been proposed as a novel sanitizer in the food and agriculture industry. In this study, we investigated the changes in the physical properties of SAEW under different conditions and the disinfection abilities of SAEW against spore-forming and non-spore-forming pathogens. Furthermore, we examined the disinfection abilities of SAEW after 12 months of shelf life on a mixed-species biofilm of *Listeria monocytogenes* Scott A and *Staphylococcus aureus*. The results showed that SAEW at 30 and 50 ppm achieved all-kill of the spore-forming pathogen *Bacillus cereus* within 30 s. Changes in the ACC and pH of the produced SAEW were generally affected by the storage conditions. Both spore-forming and non-spore-forming pathogens were not detected under treatment with 50 ppm SAEW for 5 min under HDPE-closed conditions throughout the whole storage period. Moreover, 25 mg/L SAEW can inactivate *L. monocytogenes* Scott A and *S. aureus* biofilm cells in ~2.45 and 2.57 log CFU/mL in biofilms within 5-min treatment. However, the decline of the two bacteria in the mixed-species biofilm was 1.95 and 1.43 log CFU/mL, respectively. The changes in the cell membrane permeability of the mixed-species biofilm under treatment with SAEW were observed by using atomic force microscopy and confocal laser scanning microscopy. *L. monocytogenes* Scott A was more sensitive to SAEW in the mixed-species biofilm cells. These findings exhibited strong antibiofilm activities of SAEW in impairing biofilm cell membranes, decreasing cell density, and eliminating biofilm, which suggest that SAEW is an excellent antibacterial agent in the food processing industries.

## Graphical Abstract

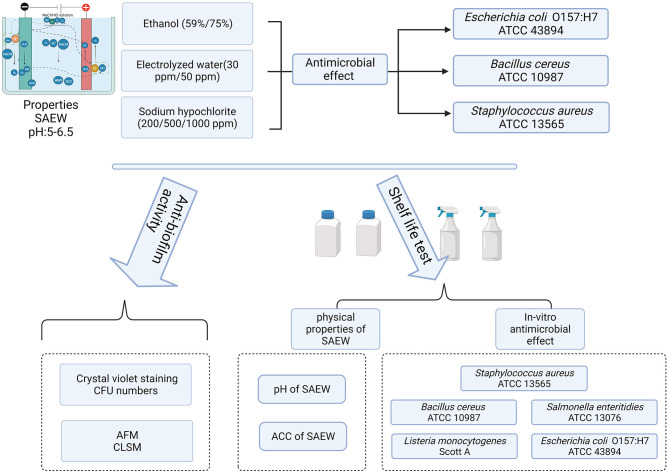

## Introduction

Biofilm is defined as a 3D extracellular matrix of microbial community adhered to the surface, which has different bacterial colonies or single types of cells in a group (Nijjer et al., 2021). This matrix composes extracellular polymeric substances, eDNA, proteins, and polysaccharides, leading to high antibiotic resistance (Karygianni et al., 2020). Biofilm is reported to relate to more than 80% of human chronic and recurrent bacterial infections and about 60% of foodborne outbreaks (Sharma et al., 2019). Although biofilms formed by single bacterial species have been extensively investigated, most microorganisms live in mixed-species biofilms, in which the growth, metabolism, and virulence genes' expressions of organisms are different from that in single-species biofilms (Hager et al., 2019; Yuan et al., 2020).

A critical concern is whether pathogenic microorganisms can be protected in the mixed-species biofilms. It has been demonstrated that the association of *L. monocytogenes* with *Pseudomonas putida* quickened the formation of the mixed-species biofilm (Whitehead and Verran, 2015), such as the presence of *P. putida* increased the resistance to benzalkonium chloride (a sanitizer) of the biofilms formed by *L. monocytogenes* (Ibusquiza et al., 2012). Indeed, biofilms in the food processing environment have been proved to contribute to the survival of foodborne pathogens in the food industry's disinfection and cleaning procedures, leading to food cross-contamination (Mazaheri et al., 2021). *Listeria monocytogenes* is a hardy gram-positive bacterium, which can form biofilms and tolerate harsh environmental conditions (Qian et al., 2020). Similarly, *Staphylococcus aureus* is a gram-positive bacterium that can cause a wide variety of chronic and acute infections, especially for the skin (Tong et al., 2015). *S. aureus* and *L. monocytogenes* both cause severe foodborne disease and are commonly found in food processing environments, including water, fish, meat, dairy, and meat (Zhao et al., 2017). Several studies have reported that the two pathogens are commonly found in polymicrobial communities on various food-contacting surfaces (Rieu et al., 2008; Zameer et al., 2010; Lee et al., 2016; Qian et al., 2020). Thus, it is necessary to explore novel antibiofilm agents to control the mixed-species biofilm of *L. monocytogenes* and *S. aureus* in the food industry.

During the past few decades, chlorine and chlorine-containing compounds have long been considered the most popular sanitizer in the food industry due to their high efficacy and relatively low cost (Wang et al., 2019). However, chlorine disinfectant, such as sodium hypochlorite (NaClO), leads to the formation of disinfection by-products, such as bromoform and chloroform (Lee and Huang, 2019). Recently, slightly acidic electrolyzed water (SAEW) has been proposed as a novel and alternative sanitizer in the food and agriculture industry (Yan et al., 2021a). SAEW is a solution produced by the electrolysis of HCl alone or in combination with NaCl in the electrolytic cell without a diaphragm (Yan et al., 2021b). SAEW exhibits a higher antimicrobial activity against foodborne pathogens, including *Salmonella* Enteritidis, *Escherichia coli* O157:H7, and *Listeria monocytogenes*, compared with the equivalent concentration of the hypochlorite ion (ClO^−^). Meanwhile, the SAEW with a slightly acidic pH (5.0–6.5) has advantages in reducing the corrosion of food processing plants, the damage to the environment, and the effect of chlorine off-gassing on human health (Ippolito et al., 2021).

Despite these advantages, the application of SAEW was limited by its instability and its high dependence on equipment. Based on these limitations, SAEW is usually freshly prepared before usage. Currently, SAEW can be used for disinfecting food, the food-connected surface, and clinical applications. Moreover, the US Environmental Protection Agency has released the recommendation list of disinfectants, including HOCl against COVID-19 (Yan et al., 2021a). Therefore, there are varieties of EW-based disinfection products on the market. However, the physical properties of SAEW and its antimicrobial activity were not thoroughly evaluated during the shelf life. Thus, it is necessary to evaluate its degradation model. Most SAEW guidelines include efficiency testing of microbial planktonic pure cultures, but little is known about their effectiveness on dual microbial biofilms (Oxaran et al., 2018).

Therefore, the aims of this study were (1) to analyze the changes in the physical properties of SAEW under different conditions (material, close, and open), (2) to evaluate the disinfection abilities of SAEW against spore-forming and non-spore-forming pathogens, and (3) to investigate the disinfection abilities of SAEW during 12 months of shelf life on a mixed-species biofilm formed by *L. monocytogenes* Scott A and *S. aureus* on food-grade stainless steel surfaces.

## Materials and Methods

### Bacterial Cultures and Growth Condition

*Bacillus cereus* (ATCC 10987), *E. coli* O157:H7 (ATCC 43895), *L. monocytogenes* Scott A (ATCC 43251), *S. aureus* (ATCC 13565), and *Salmonella* Enteritidis (ATCC 13076) were used in this study. Prior to use, the five strains were activated in brain heart infusion broth (BHI; Becton Dickinson Diagnostic Systems, Sparks, MD, USA) at 37°C for 24 h with consecutive transfers. The working concentration of bacteria was ~9 log CFU/mL.

### SAEW Preparation

The SAEW generator used in this study was supplied by Seoulin Bioscience (Seongnam, South Korea, ecoTree®). The initial SAEW was produced by electrolysis of 3% diluted hydrochloric acid solution in an electrolytic cell without membrane at a setting current of 7 A. The electrolytic cell (80 m × 12.5 mm × 0.5 T) contained both cathode (Ti) and anode (IrO_2_). Water samples were injected into the mixing tank at a 1.5 mL/min flow rate. The SAEW was collected after the amperage of generation has stabilized for 15 min.

### Procedure of Storage Experiment

Two different concentrations (30 and 50 ppm) were designed for the experiment. Changes in the ACC and pH of SAEW were tested under five different conditions, including close-HDPE-30 ppm, open-HDPE-30 ppm, close-HDPE-50 ppm, open-HDPE-50 ppm, and open-PET-50 ppm. The five samples were stored in a refrigerator at 25°C and 60% humidity for 12 months. The pH and ACC of the samples were measured at 0, 3, 6, 8, 10, and 12 months after storage. The antibacterial activities were evaluated at 0, 3, 6, 8, 10, and 12 months after storage by using *B. cereus* (ATCC 10987), *E. coli* O157:H7 (ATCC 43895), *L. monocytogenes* Scott A (ATCC 43251), *S. aureus* (ATCC 13565), and *S*. Enteritidis (ATCC 13076) broth culture.

### SAEW Treatment of Planktonic Cells

Each selected bacterial cell suspension (1 mL) was added to 9 mL of five tested SAEW samples and was shaken immediately for 1, 3, and 5 min. Then, 1 mL of each sample was transferred to a 9-mL neutralizing solution tube (0.5% sodium thiosulfate + 0.85% sodium chloride) and reacted for 1, 3, and 5 min to stop SAEW decontamination activity. Samples were serially diluted (1:10) in 9 mL of buffered peptone water (0.1% BPW; Difco, Sparks, MD, USA). The bacterial suspensions were spread onto brain heart infusion plates (BHI; Becton Dickinson Diagnostic Systems, Sparks, MD, USA). Moreover, the antimicrobial activity of SAEW (30 and 50 ppm), sodium hypochlorite (200, 500, and 1,000 ppm), and ethanol (59 and 75%) on the *E. coli* O157:H7, *B. cereus*, and *S. aureus* was tested for 30 s and 1 min by following the method of Yan et al. (2022).

### Biofilm Development

A loopful of *L. monocytogenes* Scott A (ATCC 43251) and *S. aureus* (ATCC 13565) from single colonies were cultured for 24 h at 37°C and adjusted to a concentration of 2 × 10^5^ CFU/mL. Suspensions (30 μL) of each *L. monocytogenes* Scott A or *S. aureus* and 2.97 mL TSB were incubated in 12-well microtiter plates to form single-species biofilms. Volumes of *L. monocytogenes* Scott A (20 μL) and *S. aureus* (10 μL) and a 2.97-mL BHI medium were incubated in 12-well microtiter plates to form mixed-species biofilms. The ratio of *L. monocytogenes* Scott A and *S. aureus* to form mixed-species biofilms was optimized by preexperiment data shown in Supplementary Figure 1. Sterile coupons of stainless steel (1 by 1 cm) were placed in each well of 12-well microtiter plates. The plates were then incubated at 37°C with 5% CO_2_ for 48 h.

### SAEW Treatment of Detached Biofilm

After the mature biofilms formed, the coupons were removed from the media by sterile forceps followed by rinsing with sterile 0.1% peptone water three times to remove loosely attached cells. Afterward, the mature biofilms were placed into 4 mL of the SAEW solution, which then reacted for 5 min.

### Quantification of Biofilm Adherence by Using Crystal Violet Staining

After treatment, biofilm biomass on the coupon was measured quantitatively using the crystal violet (CV) assay. In brief, the biofilm of each coupon was slightly dipped in the sterile phosphate-buffered saline (PBS, pH 7.0) three times to remove unattached cells. After drying, each coupon was stained with autoclaved 0.1% (w/v) CV solution for 30 min. The coupons were then washed and dried as described earlier, and then, the dye attached to the biofilm cell on the coupon was resolubilized with 95.0% ethanol for 45 min. The optical absorbance of the samples was measured at 600 nm using a microplate reader (Infinite 200, Tecan, Switzerland).

### Detection of Viable Cells

After treatment, the viable cells in the mixed-species biofilms were quantified using the plate counting method. In brief, each coupon was added to 9 mL of BPW, vortexed, and then diluted with BPW in subsequent 10-fold dilution. After serial dilutions, *L. monocytogenes* Scott A was spread on the selective oxford agar (DifcoTM Oxford Medium Base), and *S. aureus* was spread on the selective BPA agar (Baird–Parker agar), and then, they were incubated for 24 h at 37°C.

### Atomic Force Microscopy (AFM) and Confocal Laser Scanning Microscopy (CLSM)

The biofilm formed by *L. monocytogenes* Scott A and *S. aureus* as mono species or mixed species was further observed by AFM. Biofilm samples were fixed by adding 1 mL of 2.5% glutaraldehyde and incubating at 4°C overnight. Then, the dehydrated samples were dried in a dry oven at 50°C for 10 min. Subsequently, all samples were scanned in the tapping mode, with the scan rate and step of 1.0 Hz and 2 μm, respectively. Rq (root mean square roughness) of the captured AFM images was analyzed by Nanoscope software (version 1.7).

The mixed-species biofilm was further observed by CLSM. After treatment, the dead and live bacterial cells were stained with propidium iodide (PI) and Syto-9 (SYT), respectively, for 30 min (LIVE/DEAD BacLight™ Bacterial Viability Kit, Molecular Probes, Invitrogen). All samples were observed by the CLSM equipment (SR-CLSM; LSM880 with Airyscan, ZEISS, Oberkochen, Germany) to capture microscopic images after treatment. The 60 × objective was used to observe nucleic acid dye fluorescence excited at 488–636 nm and emitted at 504–523 nm. The biofilm images were analyzed by the ZEN 3.1, and the structural parameters of intensity were calculated (Tan et al., 2022).

### Statistical Analysis

Statistical analysis (mean values of microbial populations, ACC, and pH from each treatment and measurement) was performed using IBM SPSS Statistics Version 19 (SPSS Inc., An IBM Company, Chicago, USA). The significance of the difference was defined at *P* ≤ 0.05 using Tukey's multiple range tests.

## Results and Discussion

### Effect of SAEW on Inactivation of *E. coli* O157:H7, *B. cereus*, and *S. aureus*

**Figure 1:**
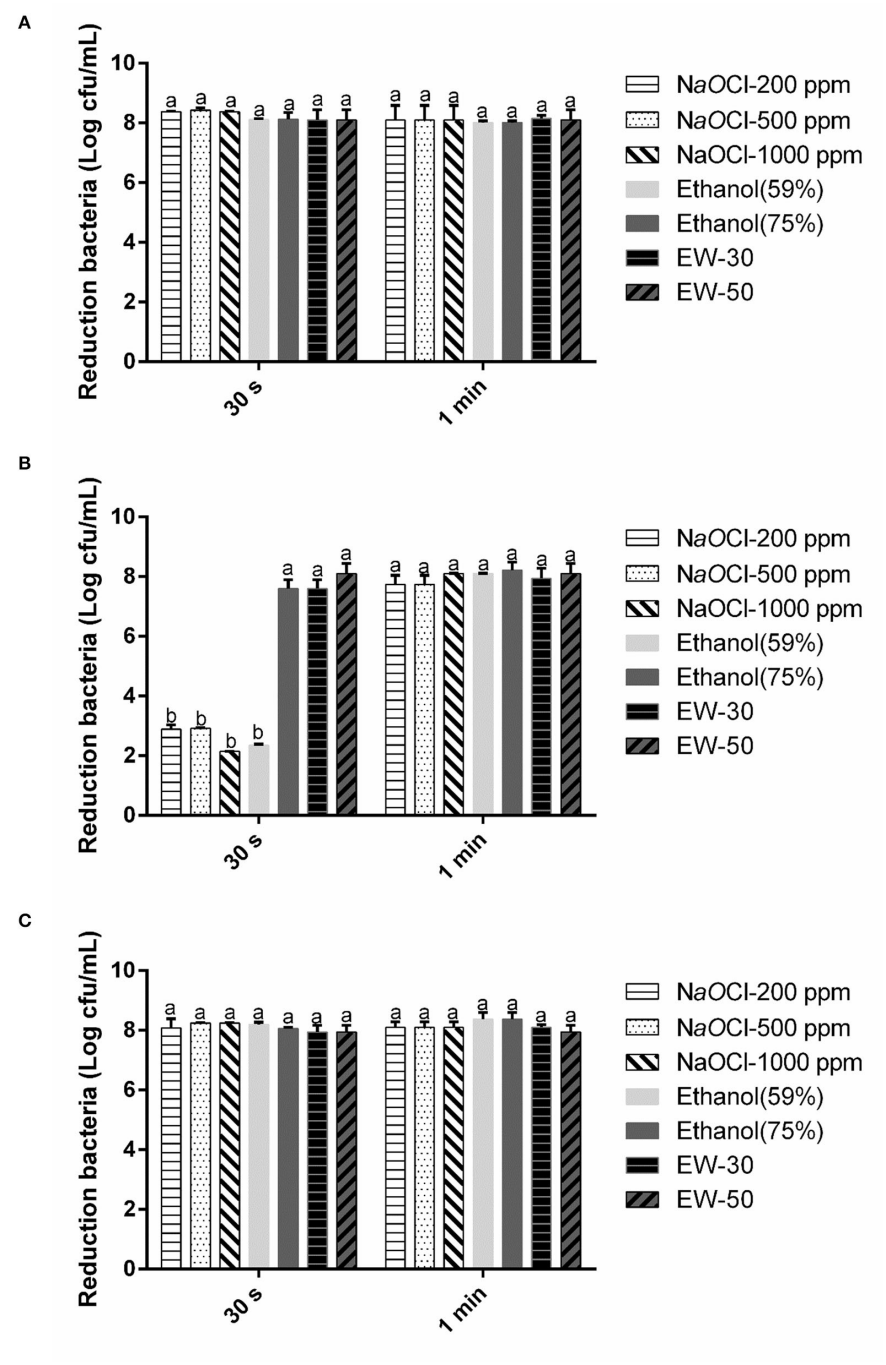
Effect of SAEW, sodium chlorite, and ethanol on *E. coli* **(A)**, *B. cereus* **(B)**, and *S. aureus* **(C)**. NaOCl, sodium chlorite. Error bars indicate the standard deviations of three measurements, and the different letters represent the significant difference (*p* ≤ 0.05).

The reductions of *E. coli* O157:H7, *B. cereus*, and *S. aureus* in pure culture treated with three different sanitizers for 30 s and 1 min are presented in Figure 1. SAEW, sodium hypochlorite, and ethanol showed a strong antimicrobial activity on the non-spore-forming pathogens in 30 s and 1 min. Whereas, SAEW achieved all-kill of the spore-forming pathogen *B. cereus* within 30 s, other disinfectant agents showed 2.14–7.62 CFU log/mL against bacteria. The reason might be that the spore-forming bacterial cytoplasm was difficult to be penetrated by most chemicals within a short time except for SAEW (Hussain et al., 2019). In addition, the difference observed between NaOCl and SAEW might be that the available chlorine in sodium hypochlorite with a pH value of 8.0 is mainly in the form of OCl^−^, while the main active gradient is HOCl (Dewi et al., 2017). It was reported that HOCl is 80–100 times more effective than hypochlorite ion (OCl^−^). SAEW showed a higher performance with a significant difference between NaOCl and ethanol, according to our results.

### Effects of Storage Condition on the SAEW Physical Properties

**Figure 2:**
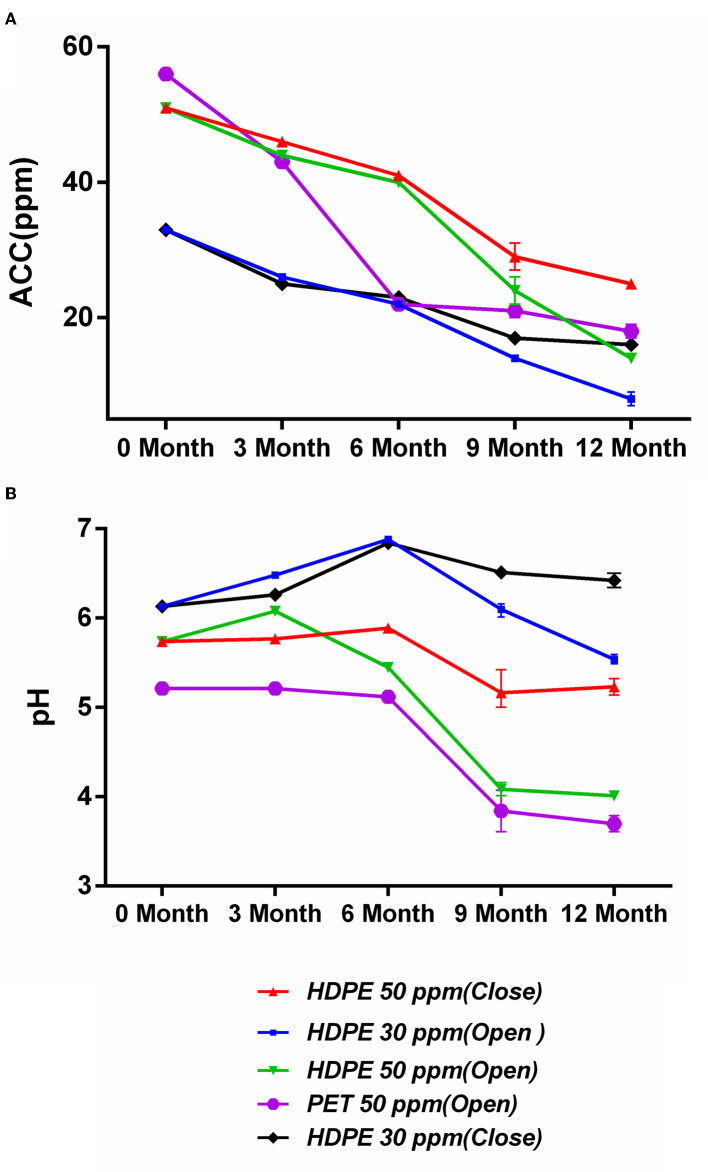
**(A,B)** Effect of storage condition on available chlorine concentration (ppm) and pH of SAEW during 12-month storage. Values are expressed as the means ± standard deviations (*n* = 3).

The degradation of the ACC of SAEW under the five different storage conditions is shown in Figure 2A. The SAEW showed a downward trend for five storage conditions during the 12-month storage period. It was found that the samples stored under closed conditions were more stable than those stored under open conditions, which is consistent with the findings of Wang et al. (2019). The closed condition can effectively inhibit the gas flow rate and reduce the volatilization rate of Cl_2_, thus inhibiting the attenuation of ACC in degree (Len et al., 2002). Furthermore, the material bottle has a significant effect on the ACC decline rate, and the degradation of ACC was slower in the HDPE bottle than in the PET bottle. The ACC of SAEW packaged with PET materials decreased significantly from 3 months, declining to 22 ppm. The reason might be that polyethylene terephthalate (PET) is clear, while high-density polyethylene (HDPE) is opaque. Some previous papers studied that light can affect the stability of ACC for chlorine-containing disinfectants (Mohammadi and Ebadi, 2021).

The changes in pH of SAEW under the five different storage conditions are shown in Figure 2B. The pH of the SAEW all sharply decreased during the 12-month storage under the opened condition, whereas it changed by only 0.29 and 0.51 for storage under the closed-50 ppm and closed-30 ppm conditions, respectively. Furthermore, under the open storage condition, the pH of the SAEW with 50 ppm decreased markedly to below 5.0, which is beyond the pH range of SAEW. In contrast, Rahman et al. reported that the pH of low-concentration electrolyzed water (LcEW) (pH 6.8; ACC 10 ppm) increased in both closed and open storage (Rahman et al., 2012). With open storage, chlorine loss followed first-order kinetics by evaporation (Quan et al., 2010). SAEW exposed to the atmosphere showed a greater reduction in oxygen and chlorine than that kept in closed condition during longer storage. In theory, the decay of ACC is the decomposition of HClO and the volatilization of Cl_2_, where deprotonation of HOCl forms OCl^−^ and a solvated H^+^ (Busch et al., 2019). In addition, the main available chlorine compound of SAEW is HClO and OCl^−^ at a pH of 5.0 to 6.5. Therein, HClO is not the main effective form of chlorine in electrolyzed water with decreased pH. Overall, the best condition in this study to maintain ACC stability and acceptable pH was the closed condition in the HDPE material bottle, declining by 50.9% of ACC at 50 ppm after 12-month storage.

### Bactericidal Activity of SAEW Under Storage Conditions

**Figure 3:**
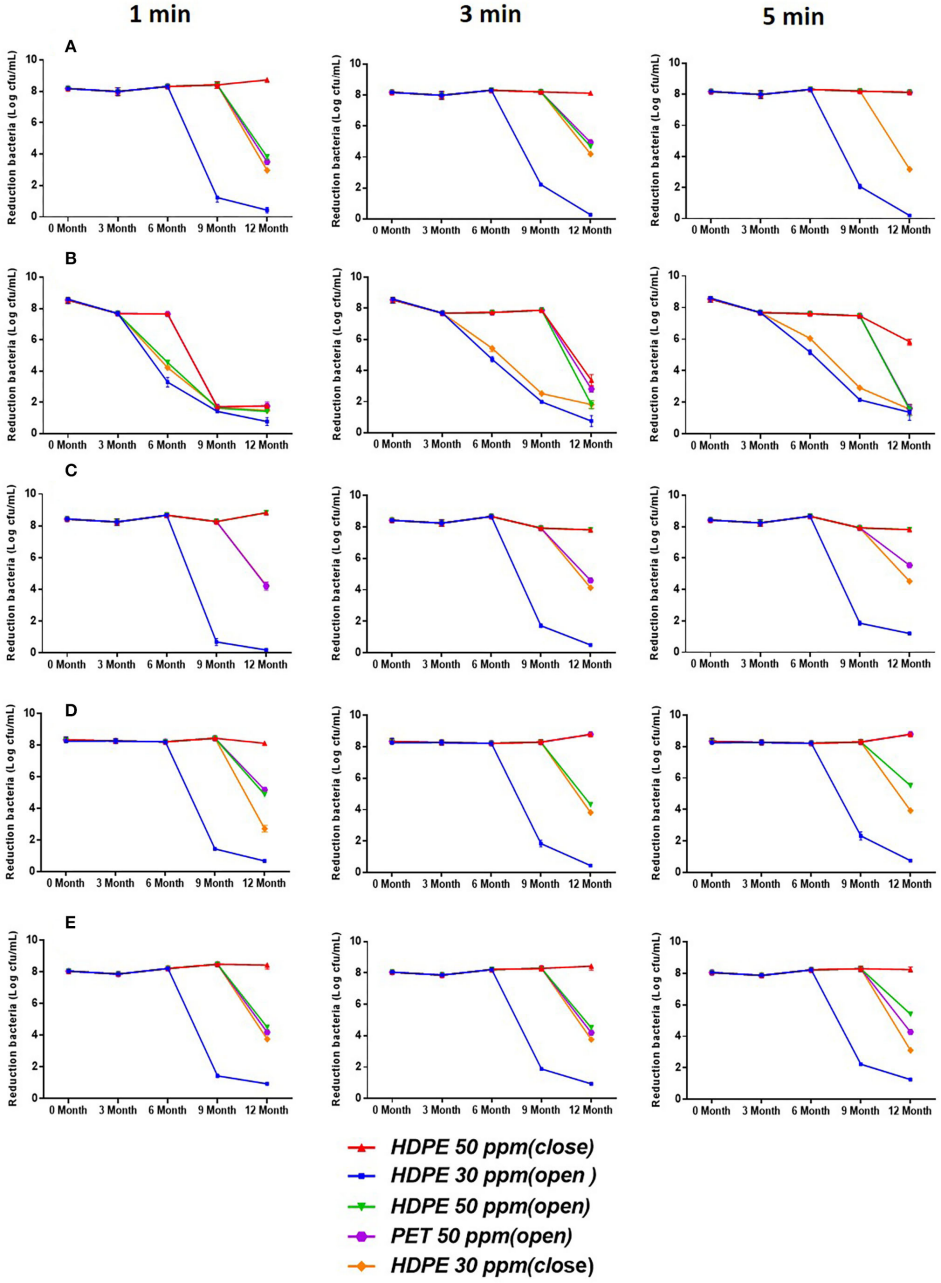
Bactericidal activity of SAEW for 1-, 3-, and 5-min treatment against *E. coli* O157:H7, *B. cereus, L. monocytogenes* Scott A, *S. aureus*, and *S*. Enteritidis during 12-month storage. **(A)** (*E. coli* O157:H7), **(B)** (*B. cereus*), **(C)** (*S. aureus*), **(D)** (*L. monocytogenes* Scott A), and **(E)** (*S*. enteritidis). Values are expressed as the means ± standard deviations (*n* = 3).

Figure 3 shows the bactericidal activity of SAEWs under opened and closed storage conditions against spore-forming bacteria (*B. cereus*) and non-spore-forming bacteria (*E. coli* O157:H7, *L. monocytogenes* Scott A, *S. aureus, S*. Enteritidis) at the different treatment times (1, 3, and 5 min). Figure 3 indicates that the storage time and the condition of SAEW have a significant effect on the reduction of the above five pathogens. SAEW under the 30 ppm-HDPE-open condition in 6 months maintained bactericidal activities against the cell culture of the five strains. However, under the 50 ppm-HDPE-closed condition, neither non-spore-forming pathogens were detectable throughout the whole storage period. These results are consistent with previous studies following Rahman and Quan (Quan et al., 2010). The chlorine loss rate of SAEW was highest when stored under an open, diffused light, and agitated condition (Rahman et al., 2016; Mohammadi and Ebadi, 2021). Rahman et al. reported that LcEW maintained bactericidal activities against cell suspensions of *L. monocytogenes* and *E. coli* O157:H7 up to 14 days under closed and 7 days under open storage conditions (Rahman et al., 2012). In addition, both ACC and dipping time have a significant effect on the reduction of *E. coli* O157:H7, *B. cereus, L. monocytogenes* Scott A, *S. aureus*, and *S*. Enteritidis. The bactericidal effect of all SAEW samples was enhanced with the increase in dipping time. Regardless of treatment time, SAEW at 50 ppm showed a significant reduction compared with SAEW at 30 ppm.

### Eradication Efficiency of SAEW Against Mixed-Species Biofilms

The interaction between different coexisting species remarkably results in the structural and functional characteristics of the multi-species biofilm (Tan et al., 2017). These multi-species interactions may be neutral, antagonistic, or communalistic, depending on the strains and the various environmental conditions (Rodríguez-Melcón et al., 2021). The mixed-species biofilm of *L. monocytogenes* Scott A and *S. aureus* was selected for further biofilm experiments based on the preliminary data (data not shown). *L. monocytogenes* and *S. aureus* can produce and adhere to biofilms on most materials and are encountered in food production plants under almost all environmental conditions.

In this study, *S. aureus* and *L. monocytogenes* were able to grow to form the single- and mixed-species biofilms on the stainless steel surfaces under the experimental conditions shown in Supplementary Figure 1. The optimization condition to form mixed-species biofilm is the ratio of 1:2 after 48-h incubation. The biovolume and cell numbers of mixed-species biofilm were greater in each single biofilm. It should be noted that *S. aureus* was the predominant species in the mixed-species biofilm. Studies by other researchers also give support for some microorganisms that can take over in the mixed-species biofilms (da Silva Fernandes et al., 2015; Oxaran et al., 2018; Qian et al., 2020).

**Figure 4:**
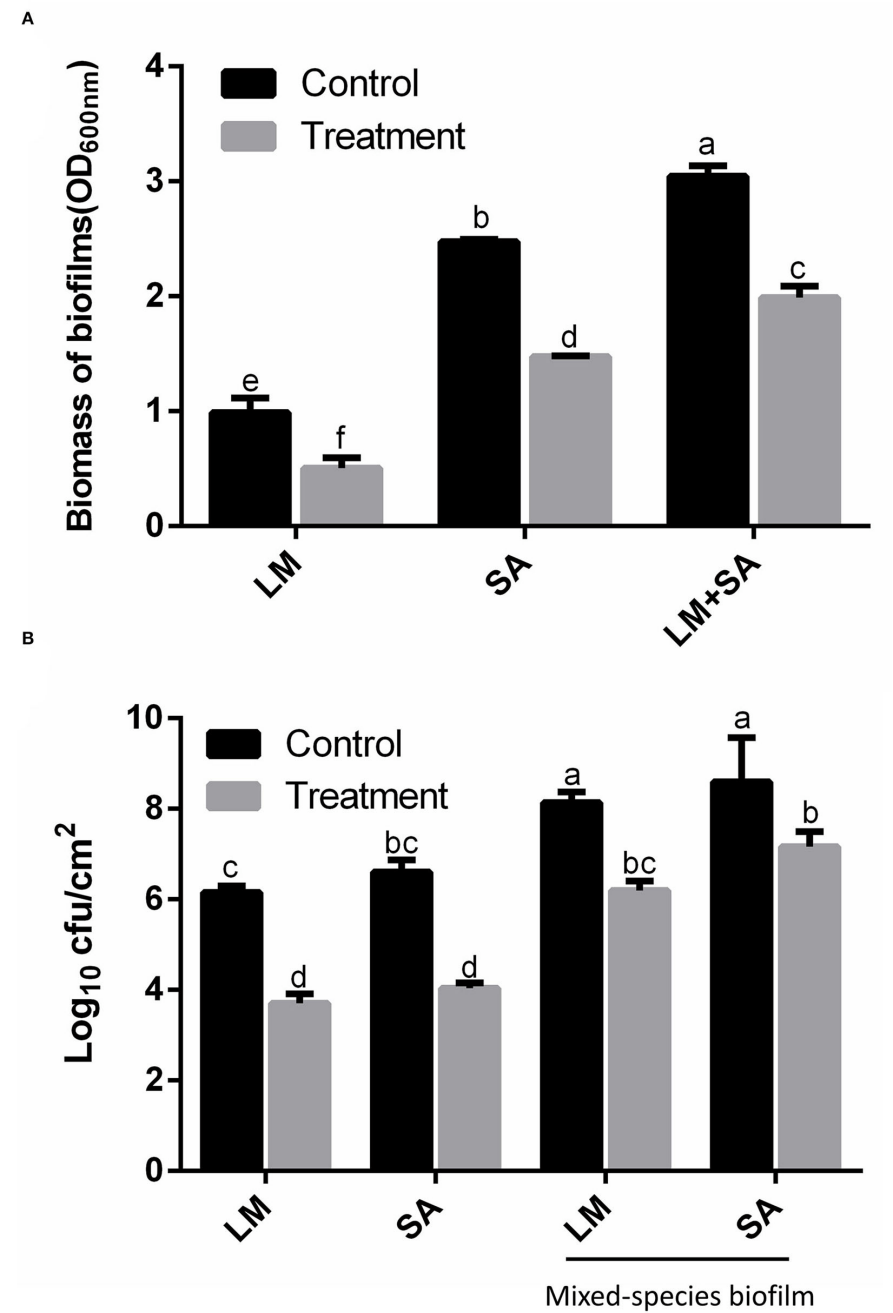
Effect of SAEW on the *Staphylococcus aureus, Listeria monocytogenes* Scott A, and mixed-species biofilms on the stainless steel surfaces. **(A)** Crystal violet staining; **(B)** CFU numbers. The plate counting method was used for the selective agar for *Staphylococcus aureus* (Baird–Parker agar) and *Listeria monocytogenes* Scott A (selective oxford agar). LM, *Listeria monocytogenes* Scott A; SA, *Staphylococcus aureus*; LM+SA, mixed-species biofilms of *Listeria monocytogenes* Scott A and *Staphylococcus aureus*. Values are expressed as the means ± standard deviations (*n* = 3). Different letters represent the significant difference (*p* < 0.05).

The effect of SAEW on the eradication potency against the mono- and mixed-species biofilm is presented in Figure 4. In Figure 4A, the biofilm biomass was determined to be 0.98, 2.46, and 3.04 (OD_600_ nm) in the *L. monocytogenes* Scott A, *S. aureus*, and the mixed-species biofilm, respectively. SAEW treatment decreased the biomass to 0.50, 1.46, and 1.99, and the decreased degree reached 48.98, 40.65, and 34.54%, respectively. Similarly, the cell numbers in the mixed-species biofilm of *L. monocytogenes* Scott A and *S. aureus* in the control group were ~8.0 log CFU/cm^2^, whose strains had the strong biofilm-forming ability. SAEW at 25 mg/L can inactivate *L. monocytogenes* Scott A and *S. aureus* in ~2.45 and 2.57 log CFU/mL in biofilms within 5 min. However, the decline of the two bacteria in the mixed species biofilm was 1.95 and 1.43 log CFU/mL, respectively. Mixed-species biofilms were more significantly resistant to SAEW than single-species biofilms after treatment with 25 mg/L SAEW (Liu et al., 2021). Therefore, SAEW still had a bactericidal effect on the mono- and mixed-species biofilms after being stored for 12 months.

### Changes in Cell Membrane Permeability of Mixed-Species Biofilm

**Figure 5:**
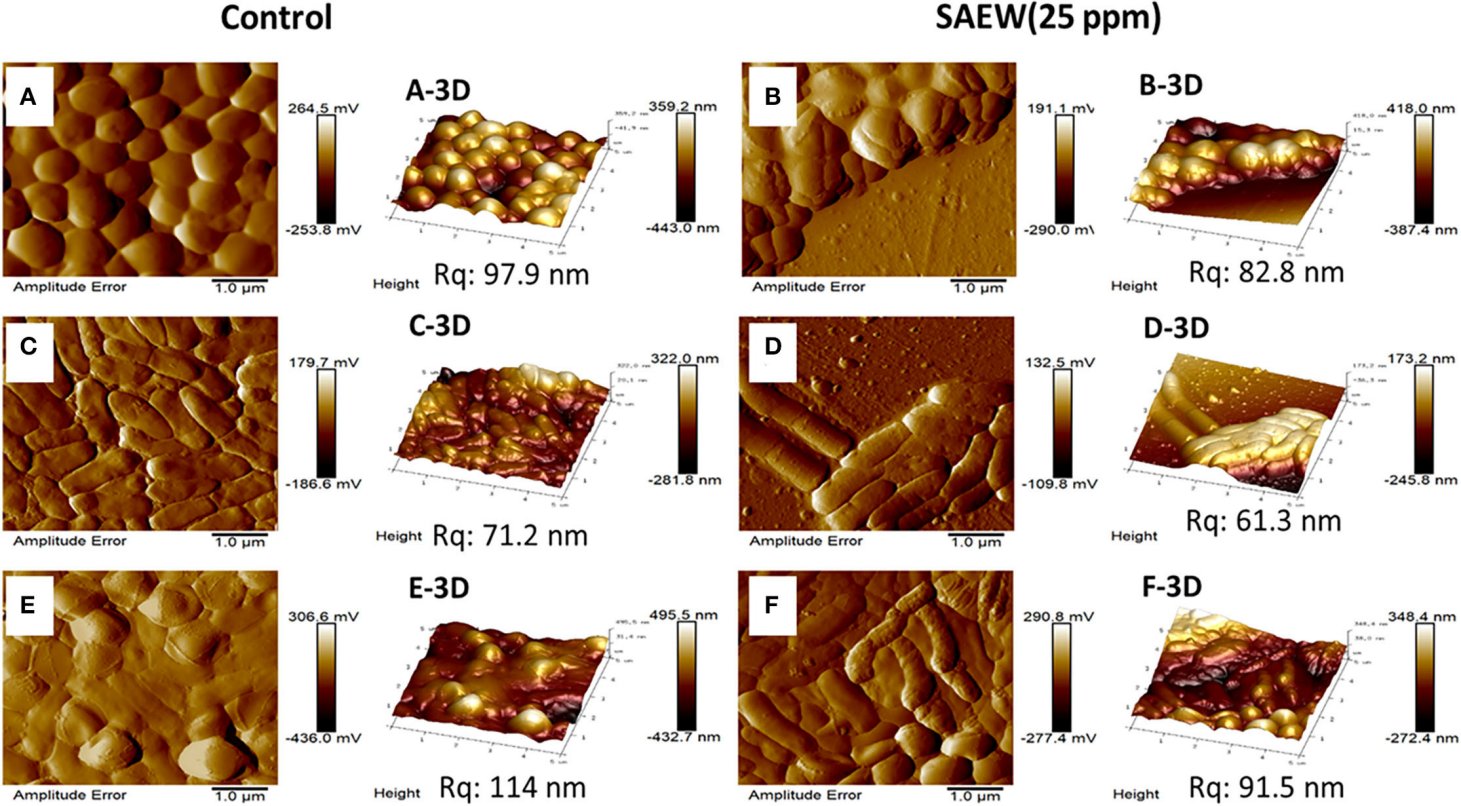
Atomic force microscopy images of the changes in the spatial structures of the single *S. aureus, L. monocytogenes* Scott A, and mixed-species biofilms under SAEW treatment. Control group [**(A)** *S. aureus*, **(C)** *L. monocytogenes* Scott A, **(E)** mixed-species biofilms] and treatment group [**(B)** *S. aureus*, **(D)** *L. monocytogenes* Scott A, **(F)** mixed-species biofilms]. Scale bar = 1.0 μm; Rq, root mean square roughness.

To further verify the effect that SAEW eradicated the mono- and mixed-species biofilms, AFM and CLSM were used to visualize the eradication efficiency of SAEW on mixed-species biofilm at the microscopic level. The *S. aureus, L. monocytogenes* Scott A, and mixed-species biofilms cells were densely packed with the biofilm thickness (359.2, 322.0, and 495.5 nm, respectively) in the control group shown in Figure 5. Notably, the cell density of single *S. aureus* and *L. monocytogenes* Scott A biofilm was decreased, and some cells began to shrink after SAEW treatment. SAEW treatment significantly reduced the average thickness of cells and biofilm, and biofilm distribution became sporadic. However, *L. monocytogenes* Scott A was more sensitive to exposure to SAEW in the mixed-species biofilms cells, in which the morphology has changed. Some authors have observed that some bacterial species such as *S. aureus* showed greater resistance to antimicrobials in mixed-species biofilms than in single-species biofilms (Oxaran et al., 2018; Carolus et al., 2019). Furthermore, the root mean square roughness (Rq) of the S. aureus, *L. monocytogenes* Scott A, and mixed-species biofilms was significantly reduced from 97.9 to 82.8 nm, 71.2 to 61.3 nm, and 114 to 91.5 nm, respectively. The Rq is one important parameter to characterize the surface texture roughness of biofilm, and its decline will significantly reduce the bacterial adhesion on the contact surface (Wu et al., 2018). Tan et al. reported that SAEW coupled with photodynamic inactivation reduced the Rq value on the surface and weakened the connection between the mixed-species biofilms (Tan et al., 2022).

**Figure 6:**
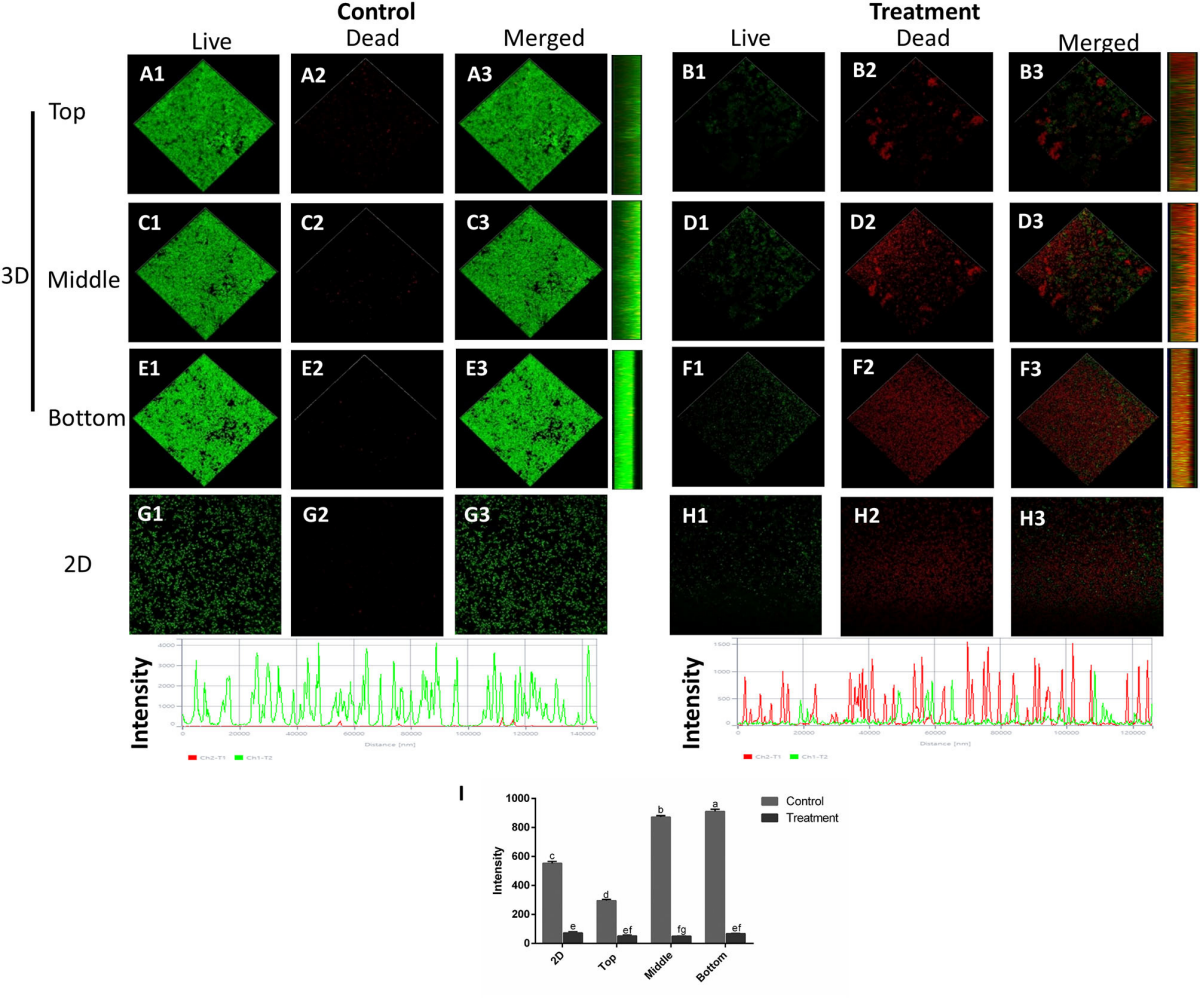
Confocal laser scanning microscopy images of the changes in the spatial structures of *S. aureus* and *L. monocytogenes* Scott A mixed-species biofilms under SAEW treatment (Scale bar = 120 μm). **(A)** (top image of control mixed-species biofilms), **(C)** (middle image of control mixed-species biofilms), **(E)** (bottom image of control mixed-species biofilms), **(B)** (top image of treatment mixed-species biofilms), **(D)** (middle image of treatment mixed-species biofilms), **(F)** (bottom image of control mixed-species biofilms), **(G)** (2D image of control biofilms), **(H)** (2D image of control biofilms), and **(I)** intensity. Different letters represent significant difference (*p* < 0.05).

Similar results were also observed in the three-dimensional structure images by using a confocal laser scanning microscope (CLSM), as shown in Figure 6. The CLSM analysis depicted live bacteria with intact membrane stained green by SYTO9 but non-viable bacteria with damaged membrane stained red by propidium iodide (Ong et al., 2019). In the control group, the mixed-species biofilm in densely populated communities is attached to the surface without membrane damage. After SAEW treatment, the biofilms were reduced and showed uneven distribution structures. Furthermore, the intensity of the green channel of mixed-species biofilm significantly decreased after 5-min of SAEW treatment. Notably, the biofilms of the top three-dimensional structures were largely reduced, and scattered structures were observed, mainly due to the strong oxidative ability of the HOCl compound directly reacting with the top layer. Several authors have observed that SAEW has the bactericidal activity against *Enterococcus faecalis* biofilms, *Vibrio parahaemolyticus* biofilms, and *Klebsiella oxytoca* biofilms (Cheng et al., 2016; Li et al., 2020; Liu et al., 2021). Based on the above results, we first confirmed the eradication effects of SAEW after a shelf life of 12 months on the mixed-species biofilm from the perspective of micro and macro levels.

## Conclusion

To our knowledge, this is the first study to assess the effects of SAEW on the mixed-species biofilms of *S. aureus* and *L. monocytogenes* Scott A during a shelf life of 12 months. The changes in physical properties and antimicrobial activity of SAEW are highly dependent on the storage time and conditions, including the material and open-closed environment. Neither spore-forming nor non-spore-forming pathogens were detectable for a 5-min reaction throughout the whole storage period under HDPE-closed conditions of 50 ppm SAEW. Furthermore, the responses of the mono-species biofilms of *S. aureus* and *L. monocytogenes* Scott A were significantly different from the mixed-species biofilm of both *S. aureus* and *L. monocytogenes* Scott A with the treatment of SAEW under 12-month storage. SAEW has strong activities to inhibit biofilm formation by preventing surface adhesions and impairing biofilm cell membrane integrities. Mixed-species biofilms were more significantly resistant to SAEW than single-species biofilms after SAEW treatment. These findings will provide a scientific basis for the research of SAEW on the antimicrobial activity and the removal of mixed-species biofilm during the shelf life.

## Data Availability Statement

The original contributions presented in the study are included in the article/Supplementary Materials, further inquiries can be directed to the corresponding author.

## Author Contributions

PY was involved in the investigation, data curation, formal analysis, and writing-original draft. RC was involved in writing-review and editing. K-hJ, XC, H-yJ, and VS were involved in the investigation. DO was involved in project administration and supervision. All authors contributed to the article and approved the submitted version.

## Funding

This work was supported by Brain Korea (BK) 21 Plus Project (Grant No. 4299990913942) funded by the Korean Government, Korea, the Collabo Project funded by the Ministry of SMEs and Startups (Grant No. C1016120-01-02), and the National Research Foundation of Korea (NRF) (Grant No. 2018007551).

## Conflict of Interest

PY, RC, K-hJ, H-yJ, and DO were employed by SeouLin Bioscience Company and Limited. The remaining authors declare that the research was conducted in the absence of any commercial or financial relationships that could be construed as a potential conflict of interest.

## Publisher's Note

All claims expressed in this article are solely those of the authors and do not necessarily represent those of their affiliated organizations, or those of the publisher, the editors and the reviewers. Any product that may be evaluated in this article, or claim that may be made by its manufacturer, is not guaranteed or endorsed by the publisher.

## Abbreviations

SAEW: Slightly acidic electrolyzed water
ACC: Available chlorine concentration
HCl: Hydrochloric acid
NaCl: Sodium chloride
HOCl: Hypochlorous acid
HDPE: High-density polyethylene
PET: Polyethylene terephthalate
*E. coli* O157:H7: *Escherichia coli* O157:H7
*B. cereus*: *Bacillus cereus*
*L. monocytogenes* Scott A: *Listeria monocytogenes* Scott A
*S. aureus*: *Staphylococcus aureus*
*S*. Enteritidis: *Salmonella* Enteritidis
CLSM: Confocal laser scanning microscopy
AFM: Atomic force microscopy
PI: Propidium iodide.
