# Supplementary material for: Stability and Antibiofilm Efficiency of Slightly Acidic Electrolyzed Water Against Mixed-Species of Listeria monocytogenes and Staphylococcus aureus
Source: Front Microbiol. 2022 May 12;13:865918. doi: 10.3389/fmicb.2022.865918 (PMC9135065; doi:10.3389/fmicb.2022.865918)
Supplement: Supplementary file 1 [file Data_Sheet_1.docx]

Supplementary Material


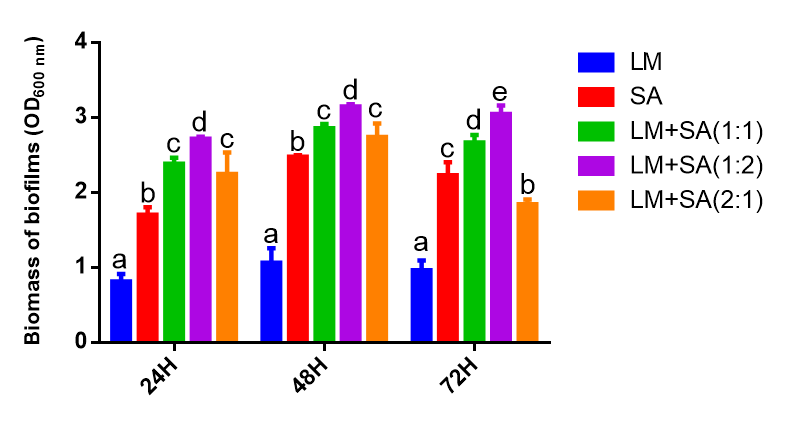


**Supplementary Figure 1.** Effect of different ratio of *L. monocytogenes* and *S. aureus* Biomass of biofilms on biomass of the mixed-species biofilm.
